# Correction: MicroRNA analysis in maternal blood of pregnancies with preterm premature rupture of membranes reveals a distinct expression profile

**DOI:** 10.1371/journal.pone.0315225

**Published:** 2024-12-04

**Authors:** Michail Spiliopoulos, Andrew Haddad, Huda B. Al-Kouatly, Haleema Saeed, Michael J. Paidas, Sara N. Iqbal, Robert I. Glazer

The fourth author’s first and last name are incorrectly switched. The correct name is: Haleema Saeed. The correct citation is: Spiliopoulos M, Haddad A, Al-Kouatly HB, Saeed H, Paidas MJ, Iqbal SN, et al. (2022) MicroRNA analysis in maternal blood of pregnancies with preterm premature rupture of membranes reveals a distinct expression profile. PLOS ONE 17(11): e0277098. https://doi.org/10.1371/journal.pone.0277098.
