# Supplementary material for: MicroRNA analysis in maternal blood of pregnancies with preterm premature rupture of membranes reveals a distinct expression profile
Source: PLoS One. 2022 Nov 3;17(11):e0277098. doi: 10.1371/journal.pone.0277098 (PMC9632843; doi:10.1371/journal.pone.0277098)
Supplement: Attachment — Submitted filename: Response to Reviewers - miRNA.docx [file pone.0277098.s002.docx]

Dear Academic Editor and Reviewers,

We greatly appreciate the quick, thorough and detailed review of our manuscript along with the very insightful comments.

Attached is our response to the comments from the Academic Editor and Reviewers.

Response to Reviewers:

Reviewer #1:

*“The results from this study have shown that there is a differential expression of specific miRNAs between PPROM and healthy controls in a small number of samples. The number of samples is very limited and due to the PPROM samples being collected at the time of hospital admission, it is not possible to determine whether these miRNAs are predictive or diagnostic of PPROM.*

*As it is not possible to distinguish the differential expression of miRNAs that could potentially lead to PPROM and those that are caused by PPROM, the bioinformatics work identifying potential targets of these miRNA markers would not be meaningful.”*

**We agree with the above reviewer comment regarding our study design not being able to differentiate between predictive and diagnostic markers for PPROM. This limitation is stated in both the “Clinical implications” as well as “Limitations” sections of the manuscript.**

**However, as noted by the title and conclusion of our study, our primary goal was to shed some light in the pathophysiology of PPROM rather than attempt to identify predictive biomarkers. Hence, we believe there is value in identifying targets for the differentially expressed miRNAs.**

**Manuscript text and conclusions have been modified accordingly.**

*“The study needs to include a validation cohort to further examine the differential expression of the identified miRNAs.”*

**We acknowledge the lack of a validation cohort in our study. Even though the criteria set for FDR in our Nanostring microarray study were very stringent there is still a possibility that qRT-PCR or other sequencing modalities might identify different expression levels for the differentially expressed miRNAs.**

**Manuscript was edited to acknowledge the above.**

*“There is important information on patient characteristics that is missing. Most of the patients were multiparous, but no information is given whether they previously had a term or preterm deliveries. It is also helpful to describe whether the cohort of patients went into spontaneous labour with PPROM or whether they were induced and some further information to explain why there is a 50% caesarean section rate for these patients.”*

**In the PPROM group, 4 patients (40%) had a history of preterm delivery compared with no patients in the control group having such history. Three patients in the PPROM group (30%) went into spontaneous labor while two (20%) were induced. Indications for c-section (50%) in the PPROM group included placenta previa (10%), malpresentation (30%) and chorioamnionitis with non-reassuring fetal heart rate pattern (10%).**

**The above information was added to the manuscript.**

***“****In addition, there is lack of information about how the samples were collected and processed. This is important to include as various factors such as phlebotomy variables, storage conditions/times, blood cell content, presence of clots/haemolysis, and processing can affect miRNA expression in serum.”*

**Blood samples were drawn from our patients on admission to the hospital, serum was isolated by centrifugation (1600g, 15 minutes at 4°C) from whole blood in less than 4 hours after collection and frozen immediately at -80 ̊C. Samples were checked for the presence of small clots and hemolysis. Specimens were maintained frozen for the duration of the study period and were shipped from Washington Hospital Center (Washington, DC, USA) directly to Georgetown University Department of Physiology (Washington, DC, USA) on dry ice where they remained blinded as to clinical outcome through testing, identified only by a unique identification number.**

**We included the above in the manuscript.**

*“Please address the Reviewer's concerns point-by-point in your response. In addition, please include the requested clinical information in the revised version, acknowledge the limitations (e.g., cause or effect of PPROM, need for a validation cohort) and adjust the conclusions accordingly.”*

**Above comments from the Academic Editor were addressed in the manuscript as well.**
